# Changes in carriage and serotype diversity of *Streptococcus pneumoniae* and other respiratory pathobionts in the UK between pre-PCV13 (2006-10), early-PCV13 (2010-12) and late-PCV13 (2012-23) periods

**DOI:** 10.1186/s41479-025-00174-y

**Published:** 2025-09-05

**Authors:** David W. Cleary, Rebecca Anderson, Jessica Jones, Rebecca A. Gladstone, Karen L. Osman, Vanessa T. Devine, Denise E. Morris, Alex J. J. Lister, Stephen Gomer, Rebecca E. Hocknell, Emily J. Dineen, Johanna M. Jefferies, James Campling, Maria Lahuerta, Kyla Hayford, Jo Southern, Bradford D. Gessner, Stephanie W. Lo, Stephen D. Bentley, Saul N. Faust, Stuart C. Clarke

**Affiliations:** 1 Department of Microbes, Infection and Microbiomes, School of Infection, Inflammation and Immunology, College of Medicine and Health, University of Birmingham, Birmingham, UK; 2 Institute of Microbiology and Infection, University of Birmingham, Birmingham, UK; 3 Faculty of Medicine, Institute for Life Sciences, University of Southampton, Southampton, UK; 4 Department of Biostatistics, University of Oslo, Oslo, Norway; 5 Parasites and microbes, Wellcome Sanger Institute, Hinxton, UK; 6 Vaccines Medical Affairs, Pfizer Ltd, Tadworth, UK; 7 Vaccines, Antivirals and Evidence Generation, Pfizer Inc, Collegeville, PA USA; 8 The Milner Centre for Evolution, Department of Life Science, University of Bath, Bath, UK; 9 NIHR Southampton Biomedical Research Centre, University Hospital Southampton Foundation NHS Trust, Southampton, UK; 10 NIHR Southampton Clinical Research Facility, University Hospital Southampton Foundation NHS Trust, Southampton, UK; 11 Global Health Research Institute, University of Southampton, Southampton, UK; 12 Infectious Disease Epidemiology Group, University of Southampton, Mailpoint 814, Level C, Sir Henry Wellcome Laboratories, University Hospital Southampton Foundation NHS Trust, South Block, Southampton, SO16 6YD UK

**Keywords:** Streptococcus pneumoniae, PCV, Carriage epidemiology

## Abstract

**Background:**

The ongoing burden of mortality and morbidity associated with *Streptococcus pneumoniae* infections requires that monitoring of carriage epidemiology continues. Here, we present data from the annual, cross-sectional surveillance study in Southampton UK on serotype epidemiology and diversity, as well as carriage of other frequent colonisers of the respiratory tract in over 7000 children over a period of seventeen years (2006–2023).

**Methods:**

Children were recruited from two sites: Site 1 - Southampton General Hospital, administered by University Hospital Southampton (UHS) NHS Foundation Trust and Site 2– a collection of community health care facilities within the Solent NHS Trust region. Recruitment was limited to children < 5-years-old. Pneumococcal serotyping was done using whole genome sequence data.

**Results:**

A total of 7,686 swabs were collected from which 2,386 (31%) pneumococci were recovered. Carriage of pneumococci has remained consistent (median carriage prevalence 31.4%) even with the almost complete removal of vaccine-type (VT) serotypes. Examining three PCV13 periods separately (pre, early and late), carriage was not significantly different at 27.7%, 35.3% and 39.3% respectively. A decrease in carriage of *Haemophilus influenzae*, *Staphylococcus aureus* and *Moraxella catarrhalis* was seen pre-PCV13 (following PCV7 implementation) but has since stabilised. Continued, low-level persistence of VTs 3, 19A and 19F was noted. PCV13 did not impact the pneumococcal serotype rank abundance despite clear reductions in targeted serotypes and fluctuations in other non-VT serotypes such as 15A, 23B and 23B1. Non-PCV13 PCV20 serotypes 10A and 11A, in addition to paired prevalence of 15B and 15C (15B/C) were in the five most isolated serotypes in the late-PCV13 period (2012 to present). Non-PCV13 PCV20 serotypes now account for approximately 40% of all carriage. By contrast, the serotypes only included in PCV15 (22F and 33F) represented just 7% in the same period.

**Conclusion:**

With consistent carriage prevalence in this UK paediatric population since PCV13 introduction, serotype epidemiology is now dominated by non-PCV13 serotypes that are in higher valency vaccines.

**Supplementary Information:**

The online version contains supplementary material available at 10.1186/s41479-025-00174-y.

## Background

The importance of monitoring community-level prevalence of respiratory pathogens has been clearly recognised in recent years. Whilst attention has shifted to viruses, pervasive challenges remain to reduce respiratory infections caused by bacterial pathogens e.g., *Streptococcus pneumoniae*. The most recent modelling estimates from the Global Burden of Disease Study indicated that *S. pneumoniae* was responsible for ~ 829,000 deaths in 2019 alone, was the main pathogen associated with lower respiratory tract infections and caused 225,000 deaths (95%CI: 180 000–281 000) in children under age 5 years [1]. Invasive pneumococcal disease (IPD) therefore remains a problem, even in countries with longstanding pneumococcal conjugate vaccine (PCV) programmes. In the UK the most recent estimates of annual childhood IPD incidences in 2022/23 were 15.88/100,000 in < 1 year-old and 8.15/100,000 in 1–4 years-old [2]. Notwithstanding the effectiveness of PCVs to prevent disease [3–8], this ongoing global disease burden requires continual vigilance.

PCVs target the primary virulence determinant of the pneumococcus, the polysaccharide capsule from which the serotype is derived and which now number over 100, with new types continuing to be described [9, 10]. A7-valent PCV (targeting serotypes 4, 6B, 9V, 14, 18C, 19F, and 23F) was introduced into the UK’s national childhood immunisation programme (NIP) in 2006 and was replaced in 2010 with a 13-valent PCV (PCV7 + additional serotypes 1, 3, 5, 6A, 7F, and 19A). Since then, there was a schedule alteration in 2020 from 2 + 1 (at age 2 months, 4 months, and 12 months) to 1 + 1 (at 12 weeks and 1 year of age). However, with recent recommendations for either PCV15 (PCV13 serotypes plus 22F and 33F) or PCV20 (PCV13 serotypes plus 22F, 33F, 8, 10A, 11A, 12F and 15B) for children in the USA and several European countries, if and when one is introduced into the UK carriage epidemiology will likely alter. Alongside this, there is the issue of serotype replacement where disease burden associated with non-vaccine serotypes increases, a phenomenon observed following both PCV7 and PCV13 introductions in some countries [11, 12], although much less so in the United States [13].

The pneumococcus is a frequent coloniser of the upper respiratory tract (URT) of children, who are considered the primary group responsible for transmission [14]. PCVs in children then are important not just for direct protection but also for indirect (herd) protection as it is well established that PCVs reduce vaccine-type colonisation [15]. As such, studies that examine pneumococcal epidemiology in this demographic, particularly in the context of changes in vaccine valency and/or schedule, are important barometers for population-level outcomes [3, 16, 17]. For example, we have previously revealed expansions of serotype 6C following PCV7 introduction and 22F following PCV13 [18, 19], in addition to continued persistence of serotype 3 linked to Clade Iα CC180 GPSC12 [20].

The URT is a diverse ecological niche, and the microbiome found there includes many bacterial species of particular interest with respect to human health [21]. For example, the pneumococcus has frequently been observed to co-colonise with *Haemophilus influenzae*, *Moraxella catarrhalis* and *Staphylococcus aureus* [22–26]. With known inter-species interactions which may shape the community of this niche [23–25, 27, 28], there is a need to consider impacts of PCVs on these additional species. Although the evidence is mixed, there are studies which report both increases of *S. aureus* and *H. influenzae* carriage following PCV introduction, with the reverse for *M. catarrhalis* [29–34]. Additionally, there is the growing body of evidence demonstrating that non-pneumococcal Streptococci (e.g., *S. mitis*) can acquire capsule biosynthesis loci and express that capsule [9]. These species are often found in the upper airways, particularly the oral cavity, with pneumococci. How these may impact our efforts to capture vaccine impact has not been fully investigated. Therefore, the wider impact of PCVs on respiratory microbiota should be examined.

Here we present follow-on data from the long-running paediatric pneumococcal carriage study in Southampton, UK. We present data on pneumococcal serotype epidemiology, serotype diversity, as well as carriage of other frequent colonisers of the child URT over a period of seventeen years in the UK. This period begins with the introduction of PCV7 into the UK NIP in 2006 and spans the PCV13 period until 2023, the verge of an era which may see the introduction of higher valency vaccines. This unique, annual, cross-sectional surveillance study has enabled us to examine the impact of PCV introductions on the URT microbiota of children < 5 years of age and provides important baseline data for consideration in future changes in vaccines and vaccination schedules.

## Methods

Paediatric Population: The population and study sites have been extensively described previously [4, 19, 35]. In brief, children were recruited from two UK sites: Site 1 - Southampton General Hospital, administered by University Hospital Southampton (UHS) NHS Foundation Trust and Site 2– a collection of community health care facilities within the Solent NHS Trust region. Site 1 recruitment has been on-going since 2006, whereas Site 2 began in 2017. Recruitment was limited to children less than five years of age. The only other exclusion criterion was that only one child per family was swabbed, irrespective of whether the child was the one attending the hospital or a sibling, and that child was swabbed only once. No exclusion criteria based on health were used. For the analysis of pneumococcal serotype diversity, an annual target of *n* = 100 isolates was determined to enable the detection ~ 50% relative reduction with 80% power at a 5% significance level.

Nasopharyngeal Swab Samples and Laboratory Processing: Nasopharyngeal Rayon Tip Transwabs (Medical Wire, Corsham, UK) in charcoal Amies media were used for swabbing and then plated onto several media (Oxoid, Basingstoke, UK) within 9 h of swabbing, including Columbia Colistin Naladixic Acid agar (CNA), Columbia Agar with Chocolated Horse Blood and Bacitracin (BAC) and Columbia Agar with Chocolated Horse Blood (CHOC). Confirmation of presumptive *S. pneumoniae* was done on CNA using optochin sensitivity indicated by a ⩾14 mm diameter inhibition zone around the disc (Thermo Scientific™, Loughborough, UK). Only one colony of *S. pneumoniae* per participant swab was selected for further analysis. *H. influenzae* was identified as characteristic colonies requiring X + V on BAC. *M. catarrhalis* was identified as Gram-negative, oxidase-positive, tributyrin-positive and DNase-positive diplococci on CHOC. *S. aureus* was identified as characteristic coagulase-positive colonies on CNA using a Pastorex Staph Plus Kit (Bio-Rad, 56353).

Pneumococcal serotyping: Isolates from skim milk, tryptone, glucose, and glycerin (STGG) stocks were cultured on CNA plates and incubated overnight at 37^o^C in 5% CO_2_ prior to DNA extraction. Extraction was carried out using QIAamp^®^ DNA mini kit (Qiagen, Hilden, Germany) according to the manufacturer’s instructions. The DNA extracts were sent to the Wellcome Sanger Institute (WSI) for whole genome sequencing (WGS) using Illumina HiSeq or 10X platforms generating initially 2 × 75 bp and later 2 × 100 bp paired-end reads from libraries prepared using TruSeq chemistry. Pneumococcal serotype was inferred using SeroBA version 1.0.5 [36].

Statistical analysis: All statistical analysis was done in R version 4.2.2 (2022-10-31) using RStudio version 2022.12.0 + 353 with graphics built using the grammar of graphics package, ggplot2 [37, 38]. Participants were characterised by whether they were recruited in the pre-PCV13, early PCV13 and late PCV13 era and were defined as having swabbing dates of 01/01/2006-30/06/2010 inclusive, 01/07/2010-30/06/2012 inclusive, and 01/07/2012 to the end of the study period (31/03/2023), respectively. Serotype diversity was calculated using *diversity*(,“simpson”) from the R package Vegan, with serotype rank-abundance model fitting done using *radfit*() from the same package.

## Results

Since the start of the study, 7,686 swabs were collected from which 2,386 (31%) pneumococci have been recovered. In total, 72.5% (5,575) of children were recruited at Site 1 (Hospital), a consequence of this being the longest running site. Here, recruitment numbers ranged from a low of *n* = 31 in 2022/23 to *n* = 542 in 2018/19. At Site 2 (community clinics), where recruitment started in 2017/18, the lowest recruitment was in 2020/21 with *n* = 228 and the highest of *n* = 470 in 2019/20. Recruitment at Site 1 has remained challenging since the SARS-CoV2 pandemic and therefore recently most children (*n* = 1,784, 60.1%) were recruited from Site 2.

Participant recruitment numbers are given in Supplementary Table 1. There was no significant difference between sites in the ratio of males to females (*p* = 0.35, row-wise z-test of proportions) with males accounting for 43% (*n* = 513/1,182) and 46% (*n* = 820/1,784) of the populations respectively at sites 1 and 2 The mean age of children recruited in site 1 was 18.1 months (± 16.37; range: 0-59.9) compared to 11.1 months (± 10.5; range: 0.4–57.1) at site 2, and this difference was statistically significant (Wilcoxon, *p* < 0.001). The median age of all children recruited was 13.0 months (IQR: 22) with 49.3% male, 42.8% female and 7.9% for whom accurate gender was not available or not recorded. We previously showed that a recent RTI (within the previous 30 days) resulted in an increased risk of carriage [35]. Whilst we do not know whether a child had an RTI at the time of swabbing, the proportion of children with a self-reported, prior cold was significantly different between the two sites (site 1 52.1% vs. site 2 46.8%, *p* < 0.001), but not for other RTIs (throat/chest infection or flu-like illness).

**Fig. 1:**
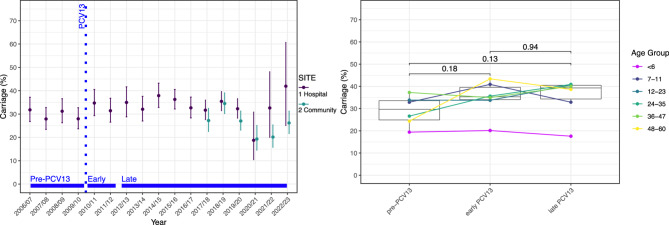
Carriage prevalence of *S. pneumoniae* across all years (left) and by PCV13 period (right). Left - Error bars represent 95% CI. From 2017/18 (year 12) onwards data has been split into Site 1 (Hospital) and Site 2 (community clinics). Combined mean carriage was 31.2%, ranging from a low of 19.2% in 2020/21 (year 15) to 37.9% in 2014/15 (year 9). Year 15 denotes a period when the UK was under various forms of non-pharmaceutical intervention (NPI). PCV13 introduction is shown as vertical dashed line; Right– Box and whisker plot showing a comparison of overall pneumococcal carriage prevalence (%) by PCV13 period. Error bars represent 95% CI. Age groups are illustrated by colour with connecting lines across PCV13 periods. No statistically significant difference was found between the pre-PCV13 and either early or late PCV13 periods

Carriage of *S. pneumoniae* between 2006/07 and 2022/23 is shown in Fig. 1. Median carriage was 31.4% (IQR: 6.7) and ranged from a low of 19.2% (95%CI 14.9–24.3) in 2020/21 to a high in 2014/15 of 37.9% (95%CI 32.8–43.2) across both sites. A lower carriage prevalence was evident in 2020/21 which corresponded to the period of non-pharmaceutical intervention introductions, e.g., lockdowns, in response to the SARS-CoV2 pandemic. As previously described this reduction was not statistically significantly [39].

Examining the three PCV13 periods (pre, early and late), carriage in the pre-PCV13 period was 27.7% (IQR: 16.5), 35.3% (IQR: 14.4) in the early-PCV13 period and 39.3% (IQR:10.0) in the late-PCV period (Fig. 1). This apparent increase was, however, not significant. Assessing carriage in age groups between these periods, we observed an increase in carriage over time in children aged 24 to 35 months, however this did not reach the significance threshold.

**Fig. 2:**
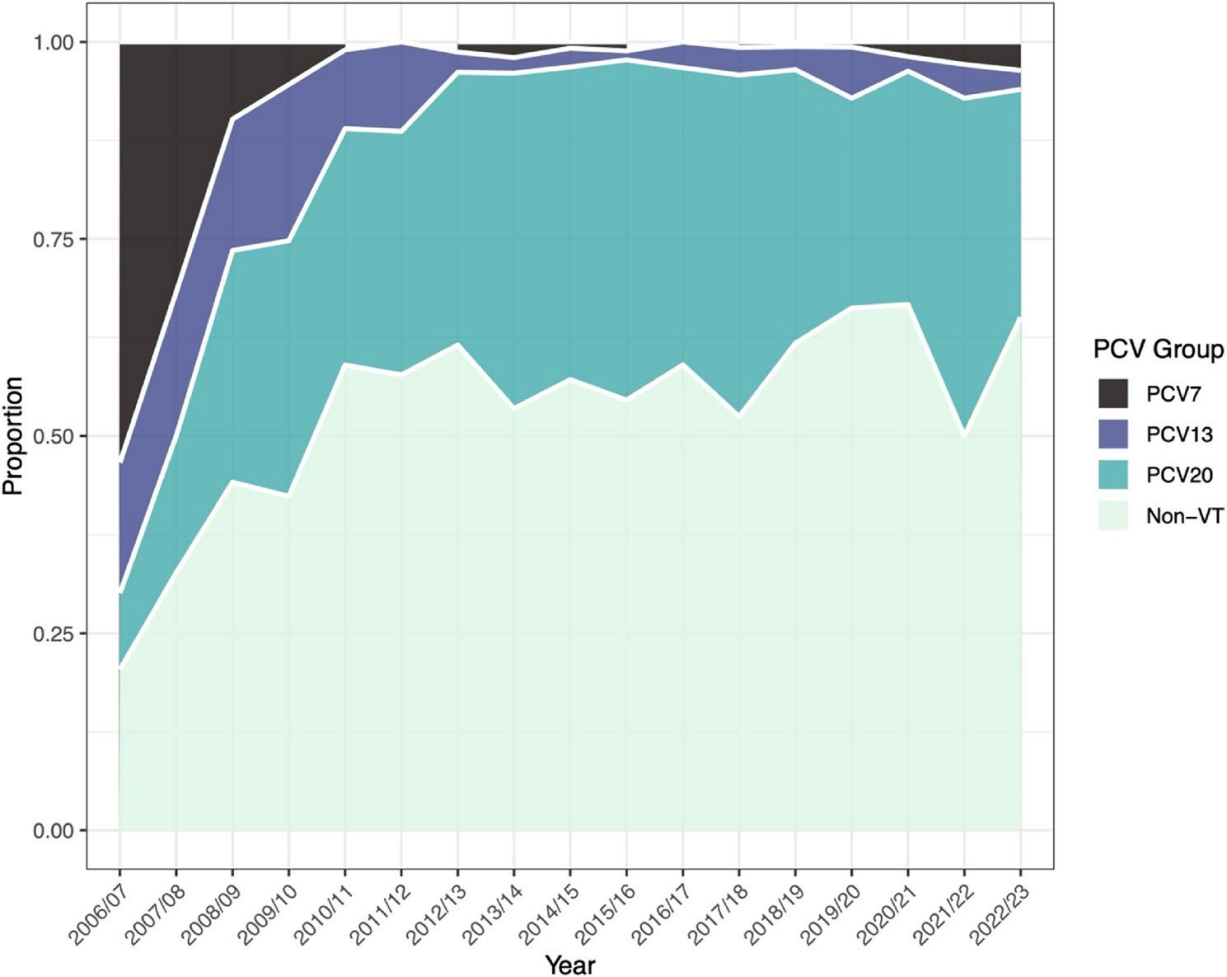
Prevalence of serotypes grouped by inclusion in PCVs. The reduction in PCV7 serotypes post-PCV7 from >50% is shown in black and of PCV13 serotypes (purple) from a high of ~20% post-PCV13. NVTs, here split to illustrate those that may be targeted by PCV20, equates to 94.0% of carriage in 2022/23

We continued to monitor the relative prevalence of pneumococcal serotypes in this paediatric survey. In total we detected 48 distinct serotypes, not including the various non-typeable genotypes that have been described. Clear decreases in both PCV7 and PCV13 serotype groups were evident, with a concomitant increase in the carriage (as a proportion of total carriage) of non-vaccine type (NVT) serotypes and those included in PCV20 (Fig. 2).

**Fig. 3:**
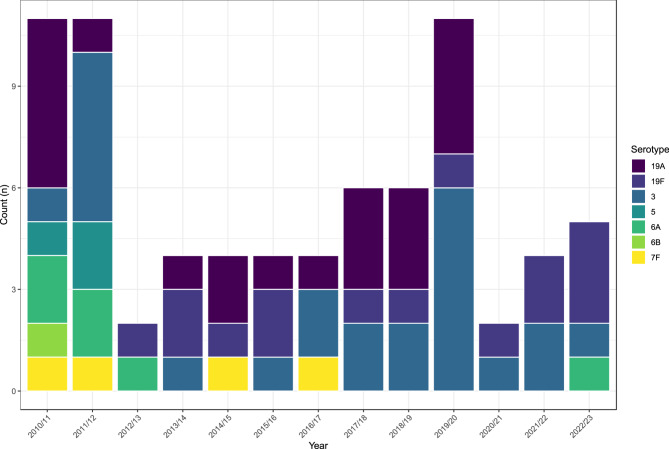
Isolation of PCV-type serotypes following PCV13 introduction in 2010. In recent years, 19F and 3 have been the most frequently isolated PCV targeted serotypes

Residual carriage of PCV7 and PCV13 serotypes persisted, though carriage was relatively low and limited to serotypes 3, 19A, 19F, 5, 6A, 6B and 7F (Fig. 3). The most frequently carried between 2010/11 (the point of PCV13 introduction) and 2022/23 (years 5 to 17) were serotypes 19A (*n*=21), 3 (*n*=24) and 19F (n=15). Serotype 6B was last detected in 2010/11 (year 5), serotype 5 in 2011/12 (year 6), and serotype 7F in 2016/17 (year 11). A single serotype 6A carriage in the last year of the study (2022/23) was the first occurrence since 2012/13 (year 7). Serotype 19A has not been detected since 2019/20 despite being relatively prevalent previously.

**Fig. 4:**
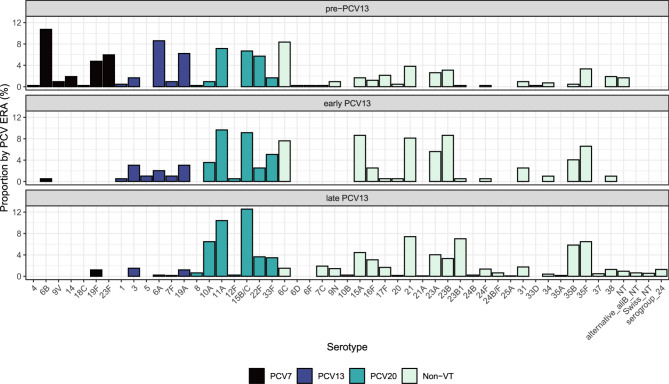
Bar plot showing serotype proportions by PCV era from the pre-PCV13 period (top) to early-PCV13 period (middle) and late-PCV13 period (bottom). Pre-PCV13: 01/01/2006-30/06/2010 inclusive; early PCV13: 01/07/2010-30/06/2012 inclusive; late PCV13: 01/07/2012 to the end of the study period. Serotypes are coloured according to vaccine type status: PCV7– black bars, PCV13– blue bars, PCV20– green, and NVT– pale green. Noticeable decreases in VT serotypes can be seen, with residual serotype 3 and 19A carriage. PCV20 serotypes 11A and 15B/C are the two most common serotypes now observed

The relative frequency of carriage of each individual serotype within PCV13 periods is shown in Fig. 4. Of those additional PCV20 serotypes it is notable that 8 and 12F were rare, however three (10A, 11A and 15B/C) were all in the five most isolated serotypes in the late-PCV13 period. Cumulatively, the most common serotypes (by carriage prevalence) across the 17 years of this study were: 11A (*n*=181, 9.8%), 15B/C (n=168, 9.1%), 21 (n=126, 6.8%), 35F (n=109, 5.9%), 10A (n=93, 5.0%) and 23B1 (*n*=91, 4.9%). In the most recent year (2022/23, year 17) 23A was the most common serotype (*n*=13, 15.5%) followed by 15B/C (n=8, 9.5%), 11A (*n*=7, 8.3%), 23B1 and 22F (both n=6, 7.1%) (data not shown).

Comparisons of serotype prevalence between the early-PCV13 and late-PCV13 period are given in Table 1. Carriage of serotypes included in PCV7 were infrequent in either period, as were serotypes 1 and 5 of PCV13. The OR for carriage prevalence of serotype 3 did not decrease, however 5, 6A and 19A all decreased significantly in the late-PCV13 period. Of the PCV20 serotypes only 22F prevalence increased significantly in the late compared to early PCV13 periods (OR: 4.60, 95%CI 1.86–11.35 *p* = 0.009). Although this was a consequence of a transient expansion on which we have previously reported [19, 40] 22F continues to be frequently isolated in carriage. This has implications for PCV15 where this serotype, along with 33F, are the only additional serotypes included. In contrast, serotype 33F was not increased between the two periods examined (OR 0.67; 95%CI: 0.333–1.361, *p* = 0.2706), and whilst 22F accounted for 7% of carriage isolates in the final year of the study, no 33F isolates were seen. Similarly to 22F, whilst a post-PCV7 expansion of 6C was previously reported [18] this increase has proved to be transient and is further reflected in the significant decreases observed between early and late-PCV13 periods reported here. Of the remaining NVTs only serotype 15A carriage prevalence changed significantly (*p* = 0.0133) in the late-PCV13 period, dropping from 8.63 to 4.42% of all serotyped isolates with an OR of carriage of 0.49 (95%CI: 0.28–0.86). Finally, previous replacement of serotype 23B by 23B1 was noted in disease surveillance [41], with an upsurge of the latter from 2011. Carriage data reflected this, where 23B decreased significantly (*p* = 0.0007) from 9% of all pneumococci in the early PCV13 period to 3% in the late. This was reflected by an increased odds of carriage for 23B1 (OR: 14.82, 95%CI 2.05–107.00, *p* = 0.0075) which in the late PCV13 period represented 7% of pneumococcal serotypes examined.

Table 1

**Table 1:** Frequency and odds ratio for serotypes isolated in the early versus late-PCV13 periods. Serotypes are grouped according to the vaccine by which they are targeted (PCV7, PCV13 and PCV20). Vaccine serotypes that were not identified in either period are not listed. *Significant *p*-values are shown in bold following Benjamini–Hochberg adjustment for FDR correction

Group	Serotype	Early PCV^§^ % (*n*)	Late PCV^§^ % (*n*)	OR (95% CI)	p*
PCV7	6B	0.51 (1)	0.00 (0)	0.05 (0.002–1.274)	0.07
	19F	0.00 (0)	1.18 (15)	4.89 (0.292–82.09)	0.2699
PCV13	1	0.51 (1)	0.00 (0)	0.05 (0.002–1.274)	0.07
	3	3.05 (6)	1.50 (19)	0.49 (0.191–1.230)	0.1275
	5	1.02 (2)	0.00 (0)	0.03 (0.002–0.646)	0.0249
	6A	2.03 (4)	0.24 (3)	0.11 (0.026–0.516)	0.0048
	7F	1.02 (2)	0.16 (2)	0.15 (0.022–1.102)	0.0624
	19A	3.05 (6)	1.18 (15)	0.38 (0.146–0.996)	0.049
PCV20	8	0.00 (0)	0.63 (8)	2.67 (0.153–46.406)	0.5007
	10A	3.55 (7)	6.48 (82)	1.88 (0.856–4.129)	0.1159
	11A	9.64 (19)	10.43 (132)	1.09 (0.657–1.809)	0.7373
	12F	0.51 (1)	0.24 (3)	0.47 (0.048–4.498)	0.5088
	15B/C	9.14 (18)	12.56 (159)	1.43 (0.856–2.384)	0.1726
	22F	2.54 (5)	3.63 (46)	4.60 (1.860–11.352)	0.0009
	33F	5.08 (10)	3.48 (44)	0.67 (0.333–1.361)	0.2706
NVT	6C	7.61 (15)	1.50 (19)	0.18 (0.092–0.370)	< 0.0001
	7C	0.00 (0)	1.90 (24)	7.79 (0.472–128.599)	0.1513
	9N	0.00 (0)	1.42 (18)	5.85 (0.351–97.518)	0.2183
	10B	0.00 (0)	0.24 (3)	1.09 (0.056–21.264)	0.9526
	15A	8.63 (17)	4.42 (56)	0.49 (0.279–0.862)	0.0133
	16F	2.54 (5)	3.08 (39)	1.22 (0.475–3.135)	0.6788
	17F	0.51 (1)	1.66 (21)	3.03 (0.442–24.718)	0.244
	20	0.51 (1)	0.16 (2)	0.31 (0.028–3.436)	0.3401
	21	8.12 (16)	7.42 (94)	0.91 (0.552–1.577)	0.7302
	21A	0.00 (0)	0.08 (1)	0.47 (0.019–11.534)	0.6425
	23A	5.58 (11)	4.03 (51)	0.71 (0.363–1.387)	0.3156
	23B	8.63 (17)	3.32 (42)	0.36 (0.203–0.652)	0.0007
	23B1	0.51 (1)	7.03 (89)	14.82 (2.053–106.997)	0.0075
	24B	0.00 (0)	0.24 (3)	1.09 (0.056–21.265)	0.9526
	24B/F	0.00 (0)	0.63 (8)	2.67 (0.153–46.406))	0.5007
	24F	0.51 (1)	1.34 (17)	2.67 (0.353–20.159)	0.3416
	Serogroup 24	0.00 (0)	1.26 (16)	5.21 (0.311–87.225)	0.2508
	25A	0.00 (0)	0.08 (1)	0.47 (0.019–11.534)	0.6425
	31	2.54 (5)	1.74 (22)	0.68 (0.254–1.915)	0.4403
	34	1.02 (2)	0.39 (5)	0.39 (0.075–2.007)	0.258
	35A	0.00 (0)	0.16 (2)	0.78 (0.037–16.328)	0.8733
	35B	4.06 (8)	5.85 (74)	1.47 (0.696–3.091)	0.3139
	35F	6.60 (13)	6.48 (82)	0.98 (0.535–1.796)	0.9485
	37	0.00 (0)	0.47 (6)	2.04 (0.114–36.003)	0.6283
	38	1.02 (2)	1.26 (16)	1.25 (0.285–5.470)	0.7689
NT	Alternative aliB NT	0.00 (0)	0.63 (8)	2.67 (0.153–46.406)	0.5007
	NT	0.00 (0)	0.95 (12)	3.94 (0.232–66.743)	0.3428
	Swiss NT	0.00 (0)	0.55 (7)	2.35 (0.134–41.347)	0.5587

§ Early PCV period: 01/07/2010-30/06/2012 inclusive; Late PCV period 01/07/2012 to the end of the study

**Fig. 5:**
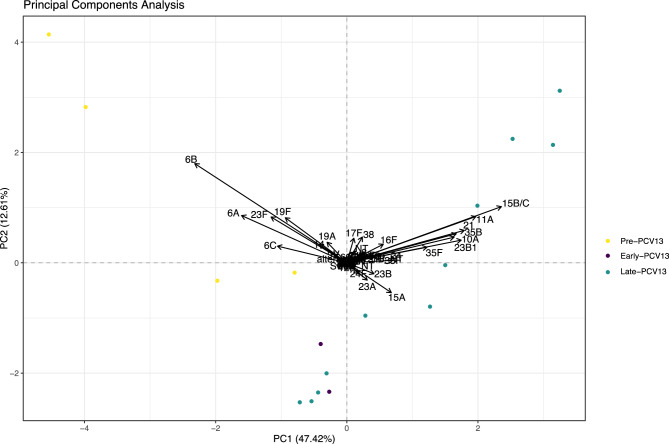
Principal Components Analysis of serotype abundance between pre-PCV13, early- and late-PCV13 periods. PCV eras are shown as coloured points with serotypes that explain the variance as arrows with the amount of variance explained indicated by arrow length. Pre-PCV13 era composition is defined by the presence of VTs 6B, 6A, 19A, 19F, 23F and the non-VT 6C

**Fig. 6:**
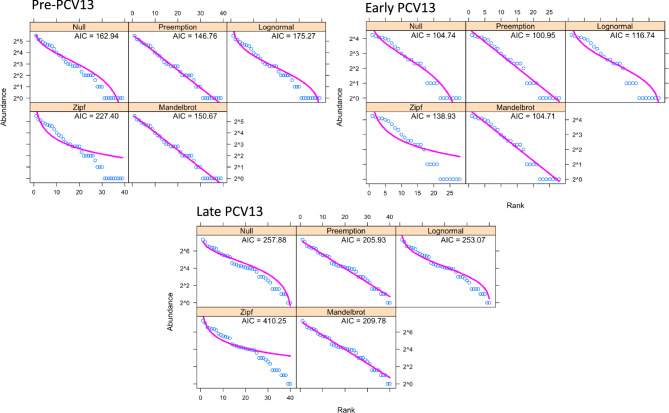
Serotype Rank-abundance distributions models in pre-, early- and late-PCV13 periods. Five models were used to examine serotype abundance. Null is equivalent to the broken-stick model. In all three the Preemption model was the best fit with the lowest AIC and suggests no disruption to population structure has occurred due to PCV13 introduction

Serotype diversity, as measured using ecological statistics which describe the composition of a community (in this case, serotypes), between pre-PCV13, early and late PCV13 periods remained unchanged, with Simpsons indexes of 0.944, 0.937 and 0.940, respectively. There was also no significant difference in the total number of serotypes observed (serotype richness), or in Shannon diversity, a measure of evenness. There was a significant difference in serotype composition between PCV13 eras (permanova, *p* < 0.001). This was driven by the removal of VT serotypes which were more prevalent in the pre-PCV13 era and their replacement with non-VTs, specifically 15A, 15B/C, 11A, 21, 35B, 10A, 23B/B1 as shown in Fig. 5. We next examined whether PCV13 introduction resulted in changes to serotype rank-abundance distributions (Fig. 6). Here we show that the niche preemption model (which fits a geometric series, or Motomura model) best described the rank abundance data for all periods examined and consequently there was no indication that serotype population structure in carriage was perturbed by PCV13 introduction.

**Fig. 7:**
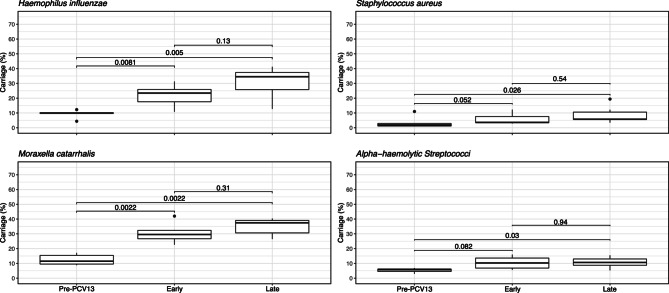
Box and whisker plots showing carriage prevalence of *Haemophilus influenzae*, *Staphylococcus aureus*, *Moraxella catarrhalis* and non-pneumococcal Alpha-haemolytic Streptococci contrasted between pre-PCV13, early- and late-PCV13 periods. Error bars show 95% CI. Significant increases in all four species are apparent between the pre-PCV13 and early-PCV13 period. No significant differences are seen between early and late-PCV13 periods

The prevalence of other respiratory pathobionts has been monitored in this cohort since 2008/09, the third year of the study and two years before the introduction of PCV13. Acknowledging that most data were collected after the introduction of PCV13 in 2010, significant increases in carriage of *Haemophilus influenzae*, *Staphylococcus aureus*, *Moraxella catarrhalis* and non-pneumococcal alpha-haemolytic Streptococci were observed in all age groups (Figure 7). However, these increases stabilised, and no significant differences were observed between the early- and late-PCV13 periods. Importantly, no increase in the proportion of methicillin-resistant *S. aureus* was observed with only 1.9% (*n*=1/52), 3.1% (*n*=1/32) and 0.7% (*n*=4/579) in the pre-, early- and late-PCV13 periods respectively.

## Discussion

The introduction of PCVs has alleviated a significant burden of disease from pneumococcal infections both in the UK and worldwide [42, 43]. Despite this success, several factors drive the requirement to continue monitoring pneumococcal carriage epidemiology. These include responses to selective pressures driven by vaccine introductions, such as capsule switching and serotype replacement, as well as the continued evolution of PCVs as valency is increased, immunogenicity is lessened for higher valency vaccines, new vaccine constructs are developed, and schedules amended (for example the switch from 2 + 1 dosing to 1 + 1 in the UK [2]). In this context, the Southampton pneumococcal carriage study, a long-running, cross-sectional, annual survey of paediatric carriage in the UK, represents a useful marker for changes in serotype epidemiology.

As reported previously, and in line with similar studies [17, 35, 44], carriage of pneumococci has remained consistent in our study since the introduction of PCV7 in 2006 even with the almost complete removal of targeted serotypes from circulation. The low-level persistence of VT-serotypes 3, 19A and 19F in carriage mirrors that of similar UK data [17] and of other European countries [45–49].

As a recognised endpoint in vaccine studies [15], carriage enables comparisons between direct and indirect impacts of schedules, as well as the opportunity to model the potential impact of alternative strategies based on new formulations [50]. Whilst serotypes 8, 12F and 9N have been previously noted for increased disease burden in England and Wales, accounting for > 40% of all-age IPD in 2016/17 [12] these are infrequently carried in the UK paediatric population, accounting for less than 3% of carriage isolates in the present study. More recently, 8 and 12F have almost completely disappeared from IPD cases in children [2]. In fact, post-pandemic (2022/23) analysis of childhood IPD in England and Wales has shown the dominance of serotypes 10A, 23B, 15B/C and 22F, which accounted for 37% of cases [2]. Except for 23B, which accounted for about a quarter, this burden is caused by serotypes that are included in PCV20. Considering the potential impact on carriage, those same serotypes, again excluding 23B, represented ~ 20% of carriage isolates in the final year of this study. The potential impact of PCV20 on both disease and carriage is therefore clear. Carriage also gives an indication of invasive potential. For example, 23B accounted for ~ 10% of IPD in 2022/23 and 7% of carriage in this study, a symmetry of prevalence observed for both 15B/C and 22F. In keeping with other studies these serotypes may therefore be considered of average invasiveness. In contrast, and similar to the highly invasive serotypes 8 and 12F, 10 A was relatively infrequent in our carriage (3.5%) in comparison to IPD incidence where it was the most common cause of invasive disease in England and Wales in 2022/23 [2], a feature that has been noted in other European countries [51].

Recent increases in serotype 7C-associated disease [52] was suggested to mirror an increase in carriage as shown in a 2017–2020 follow-up paediatric carriage study also conducted in the South of England, UK [17]. Whilst our analysis of early versus late PCV13 periods did not reveal a similar significant increase, we note that most isolates (*n* = 23/24) were from after 2016/17 (year 11), which is the year preceding the noted increase in disease. Whether this dynamic persists is an important question. As is the case with the reduction of 8 and 12F in disease, secular trends in serotype prevalence are difficult to interpret, particularly with reference to modelling the impact of higher valency vaccines [53]. Similar trends were seen with serotype 22F for example which expanded in both disease and carriage following PCV7 introduction in the UK [19, 53], before a contraction that remains unexplained. These examples only serve to underline the importance of pneumococcal carriage epidemiology for modelling disease and vaccine impacts.

Of interest is the increased carriage of other URT bacterial species shortly after the introduction of PCV13. There was concern that niche disruption or reduced fitness of replacement pneumococcal serotypes may result in the increased carriage of competitor species, particularly *S. aureus* [54]. This phenomenon was observed in post-PCV7 studies for *S. aureus* and *H. influenzae* [55] and with PCV13 [29, 56]. Whether new PCVs have similar effects remains to be seen.

There are limitations to this study. The first is that the study was conducted in a single geographic area within the UK, and in a cross-sectional manner. Therefore, extrapolation to national trends should be done with caution. Additionally, inferences regarding serotype invasiveness using carriage prevalence are made based on IPD data derived from national surveillance as no Southampton-specific IPD data with serotype information was available. Finally, carriage is being inferred from a single point-in-time isolate and therefore we are unable to examine other important features of pneumococcal carriage such as density, carriage duration or multi-serotype carriage [57, 58]. Nevertheless, the considerable strength of this work remains the nearly two decades of surveillance which has highlighted serotype expansions [18], changing genomic epidemiology of VT serotypes [20] and the impact of the SARS-CoV2 pandemic [39].

In conclusion, we have shown PCV20 serotypes accounted for approximately 40% of carriage since 2012/13, with three serotypes (10A, 11A and 15B/C) being particularly common while 8 and 12F were rarely seen. By contrast, the serotypes only included in PCV15 (22F and 33F) represented just 7% in the same period. Given these prevalences, carriage epidemiology is likely to change significantly if either vaccine is introduced, although recent data suggest PCV15 may increase overall IPD burden as the direct effects are hindered by the NVTs [53]. We highlighted rare, residual VT carriage and showed continued fluctuations in individual non-VT serotypes, most notably 15A, 23B and the genetic subtype of 23B termed 23B1. Finally, we showed that PCV13 did not impact the pneumococcal population ecology despite clear reductions in targeted serotypes, but that an immediate increase in carriage of other respiratory pathobionts did occur although has since stabilised.

## Supplementary Information

Supplementary Material 1.

## Data Availability

All sequencing data (fastqs) has been deposited in the European Nucleotide Archive under study accession PRJEB2417 (Whole genome sequencing of carried Streptococcus pneumoniae during the implementation of pneumococcal conjugate vaccines in the UK).

## Abbreviations

BAC: Columbia Agar with Chocolated Horse Blood and Bacitracin
CHOC: Columbia Agar with Chocolated Horse Blood
CNA: Columbia Colistin Naladixic Acid agar
IPD: Invasive pneumococcal disease
NIP: National immunisation programme
NVT: Non-vaccine type
PCV: Pneumococcal conjugate vaccine
URT: Upper respiratory tract
STGG: Skim milk, tryptone, glucose, and glycerin
VT: Vaccine type
