# Supplementary material for: Changes in carriage and serotype diversity of Streptococcus pneumoniae and other respiratory pathobionts in the UK between pre-PCV13 (2006-10), early-PCV13 (2010-12) and late-PCV13 (2012-23) periods
Source: Pneumonia (Nathan). 2025 Sep 5;17:20. doi: 10.1186/s41479-025-00174-y (PMC12412253; doi:10.1186/s41479-025-00174-y)
Supplement: Supplementary file 1 — Supplementary Material 1. [file 41479_2025_174_MOESM1_ESM.docx]

Supplementary Table 1: Recruitment numbers, age, gender and carriage proportion by study year. Since 2017/18 recruitment has been undertaken at both Site 1 (Hospital) and Site 2 (Community Healthcare Clinics).
